# Methanogens and Hydrogen Sulfide Producing Bacteria Guide Distinct Gut Microbe Profiles and Irritable Bowel Syndrome Subtypes

**DOI:** 10.14309/ajg.0000000000001997

**Published:** 2022-09-06

**Authors:** Maria J. Villanueva-Millan, Gabriela Leite, Jiajing Wang, Walter Morales, Gonzalo Parodi, Maya L. Pimentel, Gillian M. Barlow, Ruchi Mathur, Ali Rezaie, Maritza Sanchez, Sarah Ayyad, Daniel Cohrs, Christine Chang, Mohamad Rashid, Ava Hosseini, Alyson Fiorentino, Stacy Weitsman, Brennan Chuang, Bianca Chang, Nipaporn Pichetshote, Mark Pimentel

**Affiliations:** 1 Medically Associated Science and Technology (MAST) Program, Cedars-Sinai, Los Angeles, California, USA;; 2 Division of Endocrinology, Diabetes and Metabolism, Cedars-Sinai, Los Angeles, California, USA;; 3 Karsh Division of Gastroenterology and Hepatology, Cedars-Sinai, Los Angeles, California, USA;; 4 Vatche and Tamar Vamoukian Division of Digestive Diseases, David Geffen School of Medicine, University of California, Los Angeles, California, USA.

## Abstract

Irritable bowel syndrome (IBS) includes diarrhea-predominant (IBS-D) and constipation-predominant (IBS-C) subtypes. We combined breath testing and stool microbiome sequencing to identify potential microbial drivers of IBS subtypes.

**METHODS:**

IBS-C and IBS-D subjects from 2 randomized controlled trials (NCT03763175 and NCT04557215) were included. Baseline breath carbon dioxide, hydrogen (H_2_), methane (CH_4_), and hydrogen sulfide (H_2_S) levels were measured by gas chromatography, and baseline stool microbiome composition was analyzed by 16S rRNA sequencing. Microbial metabolic pathways were analyzed using Kyoto Encyclopedia of Genes and Genomes collection databases.

**RESULTS:**

IBS-C subjects had higher breath CH_4_ that correlated with higher gut microbial diversity and higher relative abundance (RA) of stool methanogens, predominantly *Methanobrevibacter*, as well as higher absolute abundance of *Methanobrevibacter smithii* in stool. IBS-D subjects had higher breath H_2_ that correlated with lower microbial diversity and higher breath H_2_S that correlated with higher RA of H_2_S-producing bacteria, including *Fusobacterium* and *Desulfovibrio* spp. The predominant H_2_ producers were different in these distinct microtypes, with higher RA of Ruminococcaceae and Christensenellaceae in IBS-C/CH_4_+ (which correlated with Methanobacteriaceae RA) and higher Enterobacteriaceae RA in IBS-D. Finally, microbial metabolic pathway analysis revealed enrichment of Kyoto Encyclopedia of Genes and Genomes modules associated with methanogenesis and biosynthesis of methanogenesis cofactor F420 in IBS-C/CH_4_+ subjects, whereas modules associated with H_2_S production, including sulfate reduction pathways, were enriched in IBS-D.

**DISCUSSION:**

Our findings identify distinct gut microtypes linked to breath gas patterns in IBS-C and IBS-D subjects, driven by methanogens such as *M. smithii* and H_2_S producers such as *Fusobacterium* and *Desulfovibrio* spp, respectively.


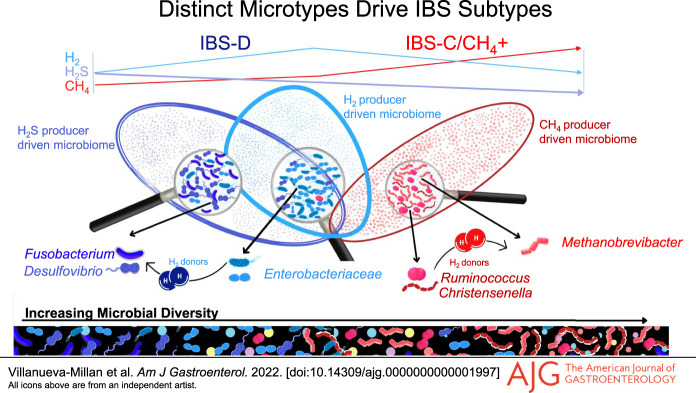


## INTRODUCTION

Irritable bowel syndrome (IBS) is estimated to affect 1 in 10 people globally, with a prevalence of 11.8%–14.0% in North America (1). IBS subjects suffer from abdominal pain, bloating, and have alterations in stool form and frequency that occur for at least 6 months (2). Based on the predominant stool pattern, IBS is divided into 3 main subtypes: constipation-predominant (IBS-C), diarrhea-predominant (IBS-D), and mixed constipation and diarrhea (IBS-M).

Although the etiology of IBS remains incompletely understood, there is a growing body of literature suggesting a role for the intestinal microbiome (3). However, specific findings have been inconsistent between studies. This may be due to the heterogeneous nature of IBS and grouping different IBS subtypes together. Despite this, one consistent finding is the association between small intestinal bacterial overgrowth (SIBO) and the IBS-D subtype, based on a recent large meta-analysis of both breath testing and small bowel culture studies (4). A diagnosis of SIBO is based on the presence of increased hydrogen (H_2_) on the breath test (5), and studies have found that SIBO is present in more than 80% of IBS-D subjects. However, new data suggest that another gas produced by gut microbes, hydrogen sulfide (H_2_S), may also be involved in diarrheal conditions. H_2_S is a gasotransmitter (gaseous signaling molecule) and is involved in numerous functions throughout the body, including inflammation and mucosal repair in the gastrointestinal (GI) tract (6). However, increased levels of sulfate-reducing bacteria (which produce H_2_S) have been linked to colorectal cancer and ulcerative colitis (7,8), which is associated with persistent diarrhea (9). More recently, we have found that increased H_2_S levels on the breath test may be associated with IBS-D (10,11), although clinically significant thresholds depend on the degree of diarrhea.

Although SIBO has been described in IBS-D, it is not associated with IBS-C. Data suggest that the gut microbiome in IBS-C is different from IBS-D as IBS-C is associated with increased methanogenesis and intestinal colonization with methanogens, now known as intestinal methanogen overgrowth (IMO) (12). Methanogens are not bacteria but are archaea, a third domain of life which lack cell nuclei and have distinct cell wall structures when compared with the other 2 domains, bacteria and eukarya (13). Within the gut, most methanogens are hydrogenotrophic, i.e., they use H_2_ generated by syntrophic bacterial species for the generation of methane (14). Interestingly, methane (CH_4_) is directly linked to slowing of intestinal transit in an animal model and an increased motility index in methane-producing IBS subjects (15) and may cause constipation (3,16).

These findings demonstrate that changes in the gut microbiome are not uniform in IBS as a whole. Rather, different microbial compositions (microtypes) may account for the differing phenotypes of IBS. Identifying these microtypes may more clearly define possible microbial pathomechanisms in IBS in general. In this study, we combine 3-gas (H_2_, CH_4_, and H_2_S) breath testing and stool microbiome sequencing to identify potential microbial drivers of clinical phenotypes in IBS.

## METHODS

### Subject recruitment

Subjects from 2 randomized controlled trials were included in this study. Baseline breath gases and stool samples from both trials were used. IBS-C subjects were recruited for a clinical trial (clinicaltrials.gov NCT03763175). IBS-C was diagnosed based on Rome IV criteria (17). Subjects were excluded if they had recent antibiotics use, had a history of loose or watery stools for >25% of their bowel movements, or had a history of laxative or enema abuse, pelvic floor dysfunction, bariatric surgery, or surgery to remove a segment of the GI tract.

The second trial recruited subjects with IBS-D (NCT04557215) based on Rome IV criteria (17). Subjects were excluded if they had a recent history of antibiotics use, previous known gastrointestinal illness, intestinal surgery, or pelvic floor dysfunction. Both trials were approved by the Cedars-Sinai Institutional Review Board, and all subjects provided written informed consent.

### Breath testing

In both studies, subjects underwent baseline (preintervention) lactulose breath testing using a system that allows the measurement of carbon dioxide (CO_2_), H_2_, CH_4_, and H_2_S (Gemelli Biotech, Raleigh, NC). Interpretation of breath test results was based on the North American Consensus for breath testing (5) and the ACG Clinical Guideline for SIBO (12). A positive H_2_ breath test was defined as a rise from baseline ≥20 ppm within 90 minutes. A positive CH_4_ breath test was defined as any measurement ≥10 ppm at any point during the test. As H_2_S is not discussed in the consensus or guideline and thresholds depend on the severity of diarrhea, H_2_S levels over the entire breath test were analyzed.

### Stool sample collection and assessment

For both studies, baseline (preintervention) stool samples were self-collected, immediately refrigerated, and then transported to the laboratory. For the IBS-C trial, only CH_4_-positive subjects provided stool samples. On arrival at the laboratory, an aliquot was transferred to an OMNIgene GUT tube (DNA Genotek, ON, Canada) and stored at room temperature before DNA extraction. Stool form was classified according to the Bristol Stool Form Scale (18).

### Stool DNA extraction

DNA extraction was performed using the MagAttract PowerSoil DNA KF Kit (Qiagen) with some modifications as described previously (19). Extracted DNAs were purified using a KingFisher Duo automated system (Thermo Fisher Scientific, Waltham, MA), and DNA purity and concentration were determined using a NanoDrop One spectrophotometer (ThermoFisher Scientific).

### Determination of stool methanogenic archaea in IBS-C and IBS-D subjects by quantitative polymerase chain reaction

Levels of 2 methanogenic archaeal species, *Methanobrevibacter smithii* and *Methanosphaera stadtmanae*, in stool from IBS-C and IBS-D subjects were determined by quantitative polymerase chain reaction (qPCR) using primers and probes targeting the beta subunit of RNA polymerase (rpoB) gene of each species (20). Assays were optimized by Applied Biosystems (Custom TaqMan Gene Expression Assays). Real-time qPCR was performed on a QuantStudio 6 Flex System (Thermo Fisher Scientific) as follows: 1 μL of 20× Custom TaqMan Gene Expression assay solution (Thermo Fisher Scientific), 10 µL of TaqMan Fast Advanced Master Mix (Thermo Fisher Scientific), 7 µL of PCR grade water, and 2 µL of template DNA (25 ng/μL) at 50 °C for 2 minutes, 95 °C for 2 minutes, 40 cycles of 95 °C for 1 second and 60 °C for 20 seconds. DNA from an *M. smithii* stock culture and from *M. stadtmanae DSM* 3091 from the Leibniz Institute Deutsche Sammlung von Mikroorganismen und Zellkulturen (DSMZ) (Braunschweig, Germany) were extracted using the same protocol, and standard curves with ten-fold serial dilutions was prepared for use as qPCR standards.

### Library preparation and 16S rRNA sequencing

Details of 16S sequencing and analysis protocols are provided in the Supplementary Digital Content (see Supplementary Information, http://links.lww.com/AJG/C678).

### Statistical analysis

The descriptive analysis is presented as mean ± SD. Categorical variables were compared with χ^2^ or Fisher exact tests, and continuous variables were compared with the *t* test or Mann-Whitney *U* test for 2 groups. Comparisons between 3 or more groups were analyzed by one-way ANOVA or Kruskal-Wallis. Correlations between variables were analyzed by Spearman rank correlation coefficients. Statistical analysis was performed using SPSS 24.0 (SPSS, Chicago, IL), SAS 9.4 (SAS Institute, Cary, NC), RStudio (RStudio, Boston, MA), and GraphPad Prism 9 (GraphPad Software, La Jolla, CA). Graph construction was performed using GraphPad Prism 9.02 (GraphPad Software). Significance was set at *P* < 0.05.

## RESULTS

### Subject demographics

A total of 171 IBS subjects were included (47 with IBS-D and 124 with IBS-C). Subjects' demographics and clinical characteristics are shown in the Supplementary Digital Content (see Supplementary Table S1, http://links.lww.com/AJG/C678). Among IBS-C subjects, 58 (47%) were CH_4_ negative (IBS-C/CH_4_−) and 66 (53%) were CH_4_ positive (IBS-C/CH_4_+) and considered to have IMO (5). A total of 8 IBS-D subjects (17%) were CH_4_ positive. IBS-C/CH_4_+ subjects were significantly older than IBS-D (*P* = 0.037) and IBS-C/CH_4_− (*P* = 0.029) subjects. No differences in sex distribution or body mass index were identified between groups (see Supplementary Table S1, http://links.lww.com/AJG/C678).

Stool form for all baseline stool samples was assessed using the Bristol Stool Form Scale. IBS-D subjects had a significantly higher average score (4.7 ± 1.27), indicating looser, more watery stools, compared with IBS-C/CH_4_+ subjects (3.2 ± 0.88) (*P* < 0.0001).

### Elevated breath H_2_ levels were more characteristic of IBS-D

**Figure 1.**
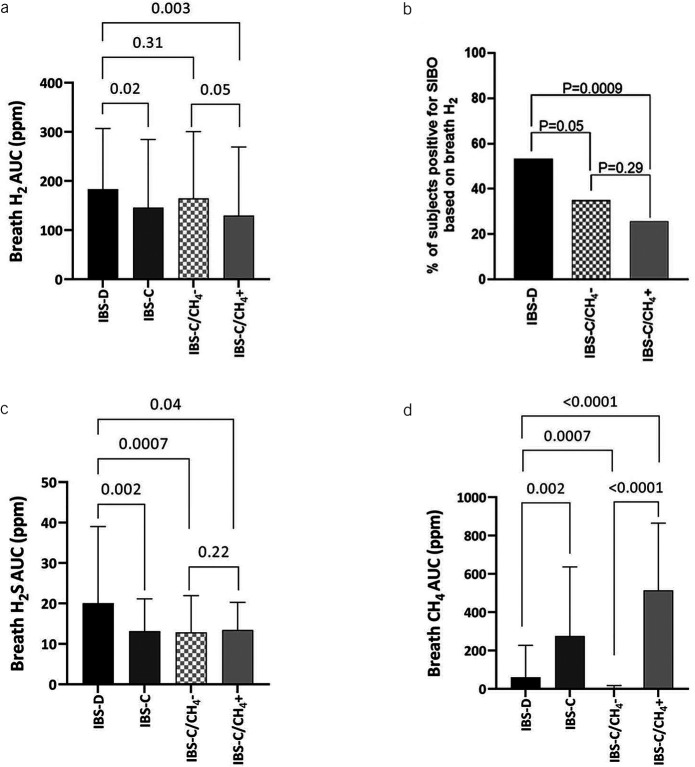
Breath test results in IBS-D and IBS-C subjects. (**a**) AUC for breath H_2_. (**b**) Rates of positivity for SIBO based on H_2_. (**c**) AUC for H_2_S. (**d**) AUC for CH_4_. Data are shown as mean ± SD. Statistical analyses by the Mann-Whitney *U* test. AUC, area under the curve; IBS, irritable bowel syndrome; IBS-C, constipation-predominant; IBS-D, diarrhea-predominant; SIBO, small intestinal bacterial overgrowth.

The area under the curve (AUC) for H_2_ was higher in IBS-D compared with all IBS-C subjects (*P* = 0.02, Figure 1a). Moreover, 53.2% of IBS-D subjects were positive for SIBO based on H_2_ (5), as were 30.1% of IBS-C subjects (*P* = 0.02). Within IBS-C, 35.09% of IBS-C/CH_4_− subjects were positive for H_2_ SIBO, compared with 25.76% of IBS-C/CH_4_+ subjects (Figure 1b). Significantly more IBS-D subjects were positive for SIBO compared with IBS-C/CH_4_+ (*P* = 0.0009, Figure 1b).

Full breath gas profiles for each group were also explored (see Supplementary Fig. S1, Fig. S2, http://links.lww.com/AJG/C678). Breath H_2_ dynamics were markedly different between IBS-D subjects and IBS-C subcategories (see Supplementary Fig. S2A, http://links.lww.com/AJG/C678). H_2_ delta values (120 minutes after lactulose ingestion vs preingestion levels) were significantly higher in IBS-D subjects vs IBS-C/CH_4_− (*P* = 0.038) and IBS-C/CH_4_+ (*P* = 0.016, see Supplementary Fig. S2B, http://links.lww.com/AJG/C678). By 15 minutes after lactulose ingestion, H_2_ levels were already higher in IBS-D vs IBS-C/CH_4_+ subjects (*P* = 0.037, see Supplementary Fig. S2A, http://links.lww.com/AJG/C678) and remained significantly higher at all time points during the breath test (Table 1).

**Table 1.**
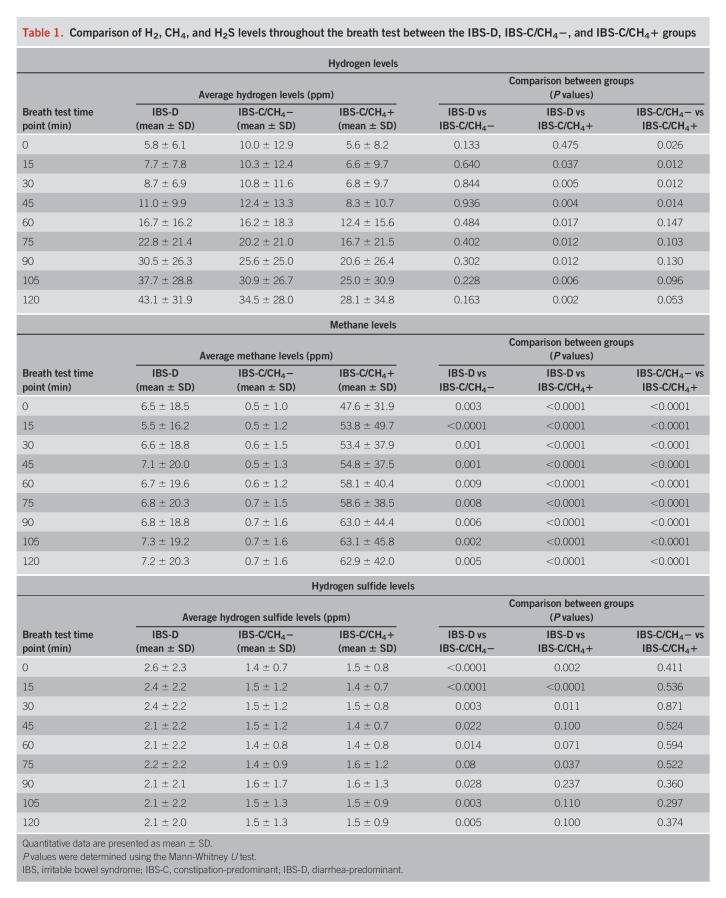
Comparison of H_2_, CH_4_, and H_2_S levels throughout the breath test between the IBS-D, IBS-C/CH_4_−, and IBS-C/CH_4_+ groups

### H_2_S levels on breath testing were also more characteristic of IBS-D

The AUC for H_2_S was also higher in IBS-D vs all IBS-C subjects (*P* = 0.002, Figure 1c). H_2_S levels were also significantly higher in IBS-D vs IBS-C/CH_4_+ subjects at 0 (*P* = 0.002), 15 (*P* < 0.0001), 30 (*P* = 0.011), and 75 (*P* = 0.037) minutes (see Supplementary Fig. S2C, http://links.lww.com/AJG/C678, Table 1).

### Elevated breath CH_4_ levels were more characteristic of IBS-C

The AUC for CH_4_ was higher in all IBS-C subjects vs IBS-D (*P* = 0.002, Table 1, Figure 1d), driven by the IBS-C/CH_4_+ group (*P* < 0.0001). CH_4_ dynamics were also different between IBS-C and IBS-D subjects (see Supplementary Fig. S1, Fig. S2, http://links.lww.com/AJG/C678), with higher CH_4_ levels in IBS-C/CH_4_+ vs IBS-D subjects at all time points during the breath test (*P* < 0.0001, see Supplementary Fig. S2D, http://links.lww.com/AJG/C678, Table 1).

### Associations between breath gases in subjects with IBS-D and IBS-C/CH_4_+

**Figure 2.**
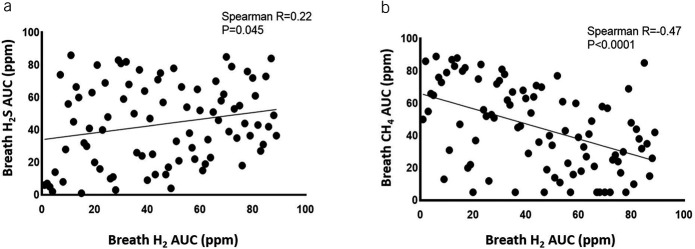
Associations between breath H_2_, H_2_S and CH_4_ in pooled IBS-D and IBS-C/CH_4_+ subjects. (**a**) Association between H_2_ and H_2_S AUCs. (**b**) Association between H_2_ and CH_4_ AUCs. AUC, area under the curve; IBS, irritable bowel syndrome; IBS-C, constipation-predominant; IBS-D, diarrhea-predominant.

In IBS-D and IBS-C/CH_4_+ subjects, breath H_2_ AUC correlated positively with breath H_2_S AUC (*R* = 0.22, *P* = 0.045, Figure 2a), but inversely correlated with breath CH_4_ AUC (*R* = −0.47, *P* < 0.0001, Figure 2b). No association was observed between breath H_2_S AUC and CH_4_ AUC (*P* = 0.9).

### Gut-derived methanogenic archaeon is associated with breath CH_4_ levels

A total of 42 IBS-C/CH_4_+ subjects and 40 IBS-D subjects provided baseline stool samples. Of IBS-C/CH_4_+ subjects, 88.09% had detectable levels of the methanogen *M. smithii* compared with 17.94% of IBS-D subjects (*P* < 0.0001). *M. stadtmanae* was less abundant in stool and was only detectable in 10% of IBS-C/CH_4_+ and 7.69% of IBS-D subjects (*P* = 1). Absolute *M. smithii* abundance correlated positively with breath CH_4_ levels, regardless of time point during the breath test, but a higher correlation coefficient was obtained using the maximum CH_4_ level reached during the breath test (*R* = 0.516, *P* < 0.0001). Absolute *M. smithii* abundance also correlated negatively with breath H_2_ levels at 105 minutes (*R* = −0.375, *P* = 0.008) and 120 minutes (*R* = −0.332, *P* = 0.02) (see Supplementary Table S2, http://links.lww.com/AJG/C678).

### IBS-D and IBS-C are characterized by distinct stool microbial signatures

**Figure 3.**
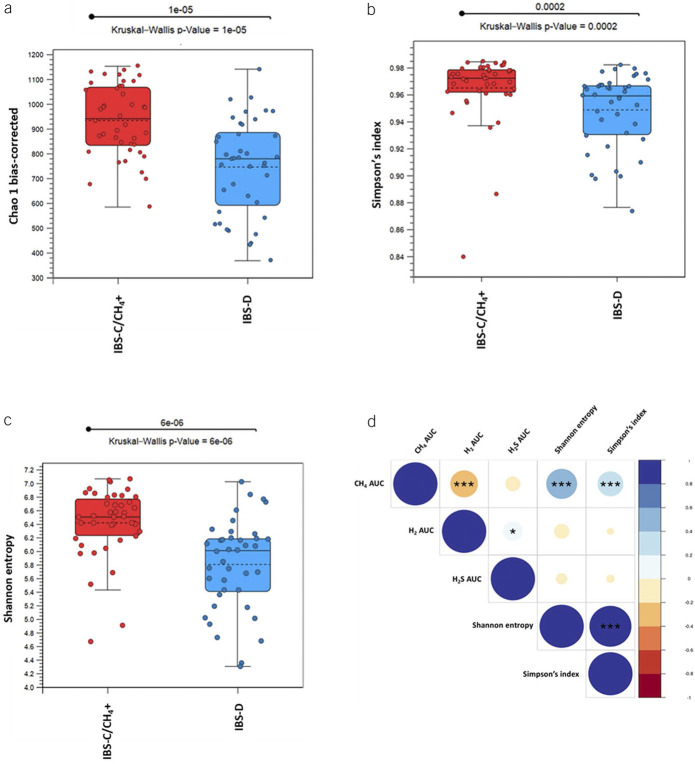
Stool microbial alpha diversity indices in IBS-D and IBS-C/CH_4_+ subjects (**a**) Chao1, (**b**) Simpson index, and (**c**) Shannon index. (**d**) Associations between microbial alpha diversity indices and breath H_2_, H_2_S, and CH_4_ (Spearman *R*). Blue gradient, positive correlations; red gradient, negative correlations. Colors indicate ranges of correlation coefficients; circle sizes denote coefficients within each range (Spearman *R*). ***P < 0.001, **P < 0.01, *P < 0.05. IBS, irritable bowel syndrome; IBS-C, constipation-predominant; IBS-D, diarrhea-predominant.

Stool samples (from 42 IBS-C/CH_4_+ and 40 IBS-D subjects) were also used for 16S rRNA sequencing. After denoising and removal of low-quality reads, a total of 3,780,543 reads were retained for taxonomic analysis (average 46,104 reads/subject). Microbial alpha diversity analysis was performed using 3 different indices, Chao1, Simpson index, and Shannon index. Chao1 is an estimator based on abundance, Simpson index gives more weight to common or dominant species (i.e., is more sensitive to species evenness), while Shannon index assumes all species (including rare species) are represented in a sample (i.e., is more sensitive to species richness) (21). This alpha diversity analysis revealed a more diverse and enriched stool microbial composition in IBS-C/CH_4_+ vs IBS-D subjects (Chao1, *P* = 1e-05; Simpson index, *P* = 0.0002; and Shannon index, *P* = 6e-06) (Figure 3a–c), resulting in distinct microbial signatures on a principal component analysis (PCA) plot (nonmetric multidimensional scaling, permutational multivariate analysis of variance [PERMANOVA] *P* < 0.001) (see Supplementary Fig. S3, http://links.lww.com/AJG/C678). Interestingly, higher breath CH_4_ AUC correlated with higher stool microbial alpha diversity (Shannon index *R* = 0.582, *P* = 1.83e-8, Simpson index *R* = 0.427, *P* = 8.7e-5, Figure 3d), whereas higher breath H_2_ AUC correlated with lower stool alpha diversity (*R* = −0.216, *P* = 0.05, Figure 3d).

Differences in microbial profiles between IBS-C/CH_4_+ and IBS-D subjects were evident even at higher taxonomic levels. The relative abundance (RA) of the archaeal phylum Euryarchaeota was higher in the stool microbiome of IBS-C/CH_4_+ subjects compared with IBS-D (fold change [FC] = 8.16, false discovery rate [FDR] *P* = 2.39E-8). Regarding bacterial taxa, the RA of phylum Firmicutes was 1.27-fold higher in IBS-C/CH_4_+ vs IBS-D (FDR *P* = 0.04), and the RA of phyla Tenericutes, Lentisphaerae, and Synergistetes were also higher in IBS-C/CH_4_+ vs IBS-D (FC = 3.47, FDR *P* < 0.0001; FC = 1.74, FDR *P* = 0.006; and FC = 2.09, FDR *P* = 0.02, respectively) (see Supplementary Fig. S4, http://links.lww.com/AJG/C678). By contrast, the RA of phyla Bacteroidetes (FC = 1.39, FDR *P* = 0.02), Fusobacteria (FC = 5, FDR *P* = 4.43E-9), Proteobacteria (FC = 1.55, FDR *P* = 0.02), Epsilonbacteraeota (FC = 2.5, FDR *P* = 3.82E-3), and Spirochetes (FC = 5.91, FDR *P* = 4.43E-9) were higher in IBS-D subjects vs IBS-C/CH_4_+ (see Supplementary Fig. S4, http://links.lww.com/AJG/C678).

**Figure 4.**
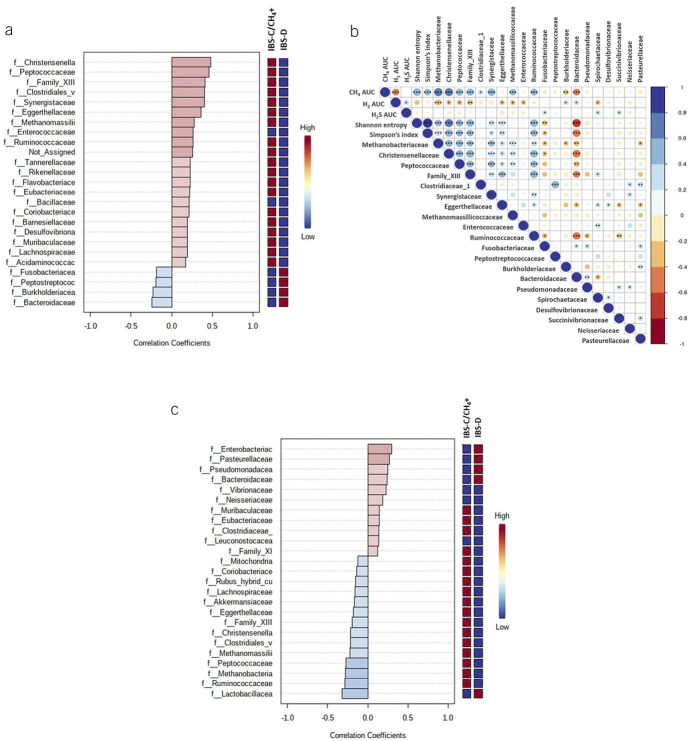
(**a**) Top 25 bacterial families correlated with relative abundance of archaeal family Methanobacteriaceae (Spearman test). (**b**) Associations between microbial alpha diversity (Shannon and Simpson indices); breath H_2_, CH_4_, and H_2_S AUC; and bacterial families associated with Methanobacteriaceae. Blue gradient, positive correlations; red gradient, negative correlations. Colors indicate ranges of correlation coefficients; circle sizes denote correlation coefficients within each range (Spearman *R*). ***P < 0.001, **P < 0.01, *P < 0.05. (**c**) Top 25 bacterial families correlated with H_2_S-producing bacterial family Fusobacteriaceae (Spearman test). AUC, area under the curve.

At the family level, the stool microbiome of IBS-C/CH_4_+ subjects was characterized by higher RA of methanogenic archaea from families Methanobacteriaceae (FC = 2.79, FDR *P* = 1.61E-6) and Methanomassiliicoccaceae (FC = 2.08, FDR = 9.16E-3) when compared with IBS-D subjects. The RA of genus *Methanobrevibacter* was higher in IBS-C/CH_4_+ subjects vs IBS-D (FC = 2.74, FDR *P* = 1.88E-5), confirming the qPCR results. Bacterial families with higher RA in IBS-C/CH_4_+ subjects vs IBS-D included Anaeroplasmataceae (FC = 7.35, FDR P3.11E-12), Flavobacteriaceae (FC = 3.84, FDR *P* = 1.48E-5), Christensenellaceae (FC = 1.91, FDR *P* = 1.91E-4), Enterococcaceae (FC = 2.92, FDR *P* = 2.16E-3), and Ruminococcaceae (FC = 1.23, FDR *P* = 0.009), among others (see Supplementary Table S3, http://links.lww.com/AJG/C678). Notably, the RA of family Methanobacteriaceae correlated positively with RA of these bacterial families in IBS-C/CH_4_+ subjects, indicating possible syntropic relationships between these microbes (Figure 4a). Finally, the RA of Methanobacteriaceae and the most important associated bacterial families (*R* > 0.25, Figure 4a) also correlated with breath CH_4_ AUC and higher stool microbial diversity (Figure 4b).

The stool microbial signature of IBS-D subjects was characterized by higher RA of several Gram-negative bacterial families, including Pseudomonadaceae (FC = 7.26, FDR *P* < 0.00001), Spirochaetaceae (FC = 5.94, FDR *P* = 5.91E-9), Fusobacteriaceae (FC = 5.23, FDR *P* = 3.75E-9), and Bacteroidaceae (FC = 1.81, FDR *P* = 4.18E-3) (see Supplementary Table S3, http://links.lww.com/AJG/C678). Most of these bacterial families negatively affected stool microbial diversity (Figure 4b). Of note, the RA of Fusobacteriaceae (which includes H_2_S-producing taxa) and of Spirochaetaceae correlated with high breath H_2_S AUC (*R* = 0.269, *P* = 0.017; *R* = 0.237, *P* = 0.035, respectively, Figure 4b). The RA of Fusobacteriaceae also correlated positively with several families that include Gram-negative bacteria, such as Enterobacteriaceae, Pasteurellaceae, Pseudomonadaceae, Bacteroidaceae, and Vibrionaceae (Figure 4c), suggesting possible relationships between these families. By contrast, Fusobacteriaceae RA inversely correlated with the families that were associated with IBS-C/CH_4_+, including Ruminococcaceae, Methanobacteriaceae, Peptococcaceae, and Methanomassiliicoccaceae (Figure 4c).

At the genus level, 35 known and unknown genera had higher RA in the stool microbiome of IBS-D subjects when compared to IBS-C/CH_4_+, including the H_2_S-producing genus *Fusobacterium* (FC = 6.08, *P* = 3.75E-12) and an unknown genus from family Desulfovibrionaceae (FC = 3, FDR *P* = 4.58E-3) (see Supplementary Table S3, http://links.lww.com/AJG/C678). There was no significant difference in RA of genus *Desulfovibrio* in IBS-D vs IBS-C/CH_4_+ subjects after *P* value correction (*P* = 0.04, FDR *P* = 0.11), but the RA of several *Desulfovibrio* OTUs was higher in IBS-D subjects vs IBS-C/CH_4_+ (FDR *P* < 0.05, see Supplementary Table S3, http://links.lww.com/AJG/C678).

### Gut microbial signatures and predicted pathways are associated with exhaled H_2_S and CH_4_ in IBS subjects

Although RA of numerous microbial taxa was different between IBS-C/CH_4_+ and IBS-D subjects (see Supplementary Table S3, http://links.lww.com/AJG/C678), microorganisms encoding enzymes necessary for H_2_S and CH_4_ production correlated with breath H_2_S and CH_4_ levels in IBS subjects. RA of *Fusobacterium* and an unknown *Desulfovibrio* species correlated positively with AUC for H_2_S (*R* = 0.33, *P* = 0.003; *R* = 0.254, *P* = 0.025, respectively). RA of genus *Methanobrevibacter* correlated positively with breath CH_4_ levels at all time points (*P* < 0.0001) and with AUC for CH_4_ (*R* = 0.658, *P* < 0.0001), consistent with the findings for stool *M. smithii* levels by PCR. Moreover, *Methanobrevibacter* RA correlated negatively with H_2_ levels at 105 minutes (*R* = −0.401, *P* < 0.001) and 120 minutes (*R* = −0.387, *P* < 0.0001).

Microbial metabolic pathway analysis further supported these associations. A signature associated with biomethanation was characteristic of the stool microbiome in IBS-C/CH_4_+ subjects and included enrichment of Kyoto Encyclopedia of Genes and Genomes (KEGG) modules associated with methane production from CO_2_, methanol, and methylamine (see Supplementary Fig. S5, http://links.lww.com/AJG/C678). The KEGG module predicting biosynthesis of F420, a cofactor used during methanogenesis (22), was also enriched in IBS-C/CH_4_+ subjects (*P* < 0.0001), and this module correlated with breath CH_4_ levels at all time points (*R* = 0.488–0.536, *P* < 0.0001) and with CH_4_ AUC (R = 0.537, *P* < 0.0001).

A biochemical signature associated with sulfur metabolism was characteristic of the stool microbiome in IBS-D subjects due to enrichment of KEGG modules associated with H_2_S production, including dissimilatory and assimilatory sulfate reduction pathways (see Supplementary Fig. S5, http://links.lww.com/AJG/C678). Although there were no direct associations between these pathways and breath H_2_S levels, the assimilatory sulfate reduction pathway correlated with H_2_ levels (*R* = 0.244, *P* = 0.027).

## DISCUSSION

In this study, we identify breath gas profiles and associated gut microtypes characteristic of different IBS phenotypes. Specifically, in IBS-C subjects with positive CH_4_ breath tests, breath CH_4_ levels were linked to higher stool levels of the methanogenic archaeon *M. smithii*, confirmed by both qPCR and sequencing. IBS-C/CH_4_+ subjects had a distinct gut microtype when compared to IBS-D, characterized by higher RA of the archaeal family Methanobacteriaceae (which includes *M. smithii*) that correlated with higher RA of specific H_2_-producing bacterial families, Ruminococcaceae and Christensenellaceae, which include known syntrophs of *M. smithii*. By contrast, IBS-D subjects were characterized by elevated breath levels of H_2_ and H_2_S. Breath H_2_S levels correlated with RA of gut bacterial H_2_S producers in IBS-D subjects, including genus *Fusobacterium* and an unknown species from genus *Desulfovibrio*, and the RA of family Fusobacteriaceae correlated with the RA of the H_2_-producing family Enterobacteriaceae. In addition, predicted microbial metabolic pathway analysis indicated enrichment of KEGG modules associated with methane production in IBS-C/CH_4_+ subjects and enrichment of KEGG modules associated with H_2_S production in IBS-D subjects. These findings suggest that increases in *M. smithii* and in bacterial H_2_S producers including *Fusobacterium* and *Desulfovibrio* species may contribute to the predominant constipation and diarrheal subtypes in IBS subjects, respectively.

Although the pathophysiology of IBS has been poorly understood, the gut microbiome seems to play a central role. Breath testing has played an important role in this because neither H_2_ nor CH_4_ is produced by human cells (23). Therefore, increased levels of these gases on the breath indicate increased gut colonization with fermenting bacteria and methanogens, respectively. The gases are then absorbed into the blood stream and excreted on exhaled breath (24). An early breath testing study suggested the importance of gut microbes in IBS (25), and 3 pivotal trials (26,27) led to the US Food and Drug Administration approval of an antibiotic treatment for IBS-D. Moreover, a higher proportion of patients with IBS-D are H_2_ positive compared with controls (4), and patients with a positive baseline H_2_ breath test are more likely to respond to rifaximin (28). These data suggest that H_2_ SIBO is part of the microbiome story in IBS-D. However, H_2_ levels do not directly correlate with diarrhea, suggesting that other microbes beyond H_2_ producers may also play a role in IBS-D.

CH_4_ on breath testing, now categorized as IMO rather than SIBO (12), was linked to IBS-C as early as 2001 (29). CH_4_ is produced by methanogenic Archaea, predominantly *M. smithii*, *Methanosphaera stadtmanae*, and *Methanomassiliicoccus luminyensis* (14), and appears to slow intestinal transit by augmenting segmental smooth muscle contractile activity in the intestinal wall (15). A more recent study suggested that CH_4_ acts through effects on enteric neurons, rather than directly on muscles (30), and proposed that CH_4_ should also be considered to be a gasotransmitter (30).

Lactulose breath testing was chosen for use in both the IBS-D and IBS-C studies in this article. Glucose is absorbed early in the small intestine, in the proximal duodenum (31), and as such can miss overgrowth in the distal small bowel (5,32,33). Although lactulose can result in false positives because of accelerated transit and colonic fermentation in some patients (5,12), it is absorbed later in the gut and therefore provides a better gas profile of a larger portion of the gut. Moreover, we previously validated that a positive lactulose hydrogen breath test (rise in hydrogen [H_2_] ≥ 20 ppm above baseline) correlated with the presence of SIBO (34) and showed that the AUC for H_2_ on the breath test correlated with predicted microbial metabolic pathways associated with energy metabolism, including formate degradation and the formation of H_2_ (34), validating that H_2_ levels on a lactulose breath test correlate with the gut microbiome.

We found a clear relationship between breath test results and the gut microbiome, with each being a predictor of IBS phenotypes. There were significant associations between CH_4_ on the breath test, gut colonization with *M. smithii*, and IBS-C. Microbial metabolic pathway analysis also revealed a correlation between the KEGG module which predicts the biosynthesis of F420 (an important coenzyme in methane production) and breath CH_4_ in IBS-C/CH_4_+ subjects. Interestingly, there was also an inverse relationship between CH_4_ and H_2_ levels, which may be consistent with the syntropic relationship between fermenting bacteria and hydrogenotrophic methanogens (14,35). Fermenting bacteria break down carbohydrates (including the lactulose or glucose substrates provided during breath testing), producing H_2_ and short-chain fatty acids. However, accumulation of H_2_ beyond certain levels inhibits bacterial growth. Hydrogenotrophic methanogens such as *M. smithii* use H_2_ to generate CH_4_ (14,35), reducing H_2_ levels and increasing CH_4_ levels. The reduction in H_2_ in turn facilitates continued growth of the fermenting bacteria, benefiting both syntrophs.

Another interesting finding is that IBS-C/CH_4_+ subjects have greater gut microbial diversity than IBS-D. Perhaps reducing localized H_2_ concentrations allows specific syntrophic bacterial populations to proliferate, thus increasing diversity. The bacterial genera which co-occur most with methanogens are *Christensenella*, *Bacteroides*, *Ruminococcus*, and *Desulfovibrio* (35). Consistent with this, we found higher RA of H_2_-producing families Christensenellaceae and Ruminococcaceae which correlated with RA of Methanobacteriaceae in IBS-C/CH_4_+ subjects. Moreover, Ruaud et al. (36) demonstrated that *Christensenella* spp can transfer H_2_ to *Methanobrevibacter* spp, confirming the syntrophic relationship between these species.

We also identified higher breath H_2_S and greater abundance of specific H_2_S-producing bacteria in IBS-D subjects compared with IBS-C/CH_4_+, including the family Fusobacteriaceae. Moreover, higher RA of Fusobacteriaceae correlated with higher RA of the H_2_-producing family Enterobacteriaceae, as well as other families that include Gram-negative bacteria such as Pasteurellaceae, Pseudomonadaceae, Bacteroidaceae, and Vibrionaceae, and with lower RA of the bacterial families associated with Methanobacteriaceae, such as Christensenellaceae and Ruminococcaceae. These findings illustrate that the microbial profiles in IBS-D and IBS-C/CH_4_+ subjects are very distinct. H_2_S-producing bacteria compete with methanogens for H_2_ in the gastrointestinal tract (37), and consistent with this, we found negative correlations between RA of *Fusobacterium* spp and *M. smithii* and between *Fusobacterium* spp and AUC for CH_4_.

Microbial metabolic pathway analysis also identified enrichment of KEGG modules associated with H_2_S production, including dissimilatory and assimilatory sulfate reduction pathways, in IBS-D subjects. These findings are consistent with our previous findings linking diarrhea and breath H_2_S levels (10) and independent studies linking H_2_S to diarrheal disorders including ulcerative colitis (38). Supporting this, rat studies have shown that H_2_S acts as a smooth muscle relaxant, possibly through direct inhibition of L-type calcium channels (39). We also found higher RA of genus *Fusobacterium*, which includes H_2_S producers, and an unknown *Desulfovibrio* species in IBS-D subjects, as well as correlations between *Fusobacterium* and AUC for H_2_S. These data suggest that *Fusobacterium* and *Desulfovibrio* spp may drive H_2_S production in IBS-D and thus contribute to the predominant symptom of diarrhea.

This study has some limitations. As stool was not obtained from IBS-C/CH_4_− subjects, it was not possible to determine microbial composition in this group, nor did we have healthy controls. It is interesting that the IBS-C/CH_4_− subjects had even lower CH_4_ levels than in the IBS-D group and had similar H_2_ levels. All IBS-C subjects were required to meet extensive inclusion and exclusion criteria before undergoing breath testing, lessening the possibility that differences in concomitant diseases and medications contribute to the differences in IBS-C/CH_4_− vs IBS-C/CH_4_+ subjects, although information was not obtained for other potential confounders such as diet or smoking. Determining the microbiome composition in IBS-C/CH_4_− subjects, and whether they may in fact constitute a different IBS-C microtype with different causal microbes, will be important to determine in future studies. In addition, although this work focused on the importance of CH_4_, H_2_, and H_2_S and corresponding microbial producers in driving the predominant symptoms in IBS, other factors may need further exploration. For example, we found increased RA of family Spirochaetaceae in IBS-D vs IBS-C/CH_4_+, and 1 study found an increase in *Brachyspira* abundance in subjects with IBS, associated with colonic eosinophil counts (40). How this finding factors into gas dynamics remains unexplored.

In conclusion, our data identify distinct gut microtypes linked to breath gas patterns in subjects with IBS-C and IBS-D. IBS-C subjects are characterized by detectable breath CH_4_, linked to higher colonization with methanogenic archaea (predominantly *M. smithii*) and a constipation phenotype. By contrast, IBS-D subjects are characterized by higher breath H_2_ and by higher breath H_2_S linked to increased prevalence of H_2_S-producing bacteria (predominantly *Fusobacterium* and *Desulfovibrio* spp) and a diarrhea phenotype. Furthermore, the predominant H_2_ producers were different in these distinct microtypes, with higher RA of Ruminococcaceae and Christensenellaceae in IBS-C/CH_4_+ and higher Enterobacteriaceae RA in IBS-D. Identification of these distinct microtypes may facilitate a better understanding of the relationship between the gut microbiome and the heterogeneous phenotypes of IBS and allow us greater precision in the development of targeted microbiome-based therapies.

## CONFLICTS OF INTEREST

**Guarantor of the article:** Mark Pimentel, MD.

**Specific author contributions:** Conceptualization: M.P., R.M. Formal analysis: G.L., M.J.V.M., J.W., A.R., M.P. Methodology: M.J.V.M., G.L., W.M., S.W., M.P. Investigation: M.J.V.M., G.L., G.P., M.L.P., G.M.B., M.S., S.A., D.C., S.W., C.C., M.R., A.H., A.F., B.C., N.P., A.R., M.P. Visualization: M.J.V.M., G.L. Funding acquisition: G.B., R.M., M.P. Project administration: R.M., M.P. Supervision: W.M., S.W., C.C., M.R., R.M., M.P. Writing–original draft: M.J.V.M., G.L., G.B., W.M., J.W., M.P., Writing–review & editing: M.J.V.M., G.L., G.B., W.M., J.W., A.R., R.M., M.P.

**Financial support:** This study was supported in part by funds from The Monica Lester Charitable Trust (R.M.), The Elias, Genevieve, and Georgianna Atol Charitable Trust (R.M.), Synthetic Biologics, Inc (A.R.), Bausch Health (M.P.) and The National Philanthropic Trust (M.P.).

**Potential competing interests:** M.P. is a consultant for Bausch Health, Ferring Pharmaceuticals Inc., and Vivante Health Inc. M.P. has received grant support from Bausch Health and Synthetic Biologics. R.M. has received grant support from Valeant Pharmaceuticals. A.R. is a consultant/speaker for and has received grant support from Bausch Health. In addition, Cedars-Sinai Medical Center has licensing agreements with Bausch Health and Gemelli Biotech. A.R., M.P., and R.M. have equity in Gemelli Biotech and M.P. has equity in Synthetic Biologics. All other authors report no conflicts of interest.

**Data availability:** The data sets generated during the current study are available in the National Center for Biotechnology Information (NCBI) BioProject Repository https://www.ncbi.nlm.nih.gov/bioproject under BioProject PRJNA804225.

**Study Highlights**

**WHAT IS KNOWN**

✓ Irritable bowel syndrome (IBS) includes diarrhea-predominant (IBS-D) and constipation-predominant (IBS-C) subtypes.
✓ The gut microbiome is associated with IBS, but the roles of specific gut microbial populations are poorly understood.
✓ The gases hydrogen (H_2_), hydrogen sulfide (H_2_S), and methane (CH_4_) are produced by gut microbes.
✓ Increased CH_4_ on the breath test is associated with IBS-C and correlates with increased predominance of methanogens, including *Methanobrevibacter smithii*.
✓ H_2_ levels do not directly correlate with diarrhea, but H_2_S has recently been linked to a diarrhea phenotype.

**WHAT IS NEW HERE**

✓ Distinct gut microtypes are linked to breath gas patterns in IBS-C and IBS-D subjects.
✓ In CH_4_+ IBS-C subjects, increased breath CH_4_ correlated with increased gut microbial diversity.
✓ In IBS-D subjects, increased breath H_2_ correlated with lower microbial diversity and increased breath H_2_S correlated with increased predominance of H_2_S producers, including *Fusobacterium* and *Desulfovibrio* species.
✓ The predominant H_2_ producers in IBS-C subjects were Ruminococcaceae and Christensenellaceae, which include known bacterial syntrophs for methanogens.
✓ Predicted microbial metabolic pathway analysis indicated enrichment of pathways associated with methanogenesis in IBS-C/CH_4_+ subjects and enrichment of pathways associated with H_2_S production in IBS-D subjects.

## Supplementary Material

SUPPLEMENTARY MATERIAL
