# Supplementary material for: Methanogens and Hydrogen Sulfide Producing Bacteria Guide Distinct Gut Microbe Profiles and Irritable Bowel Syndrome Subtypes
Source: Am J Gastroenterol. 2022 Sep 6;117(12):2055–66. doi: 10.14309/ajg.0000000000001997 (PMC9722381; doi:10.14309/ajg.0000000000001997)
Supplement: SUPPLEMENTARY MATERIAL [file acg-117-2055-s001.pdf]

Supplemental Information

Supplemental Methods

Library preparation and 16S rRNA sequencing

16S rDNA hypervariable V3 and V4 region libraries were prepared following the Illumina (Illumina, San Diego, CA) protocol (https://support.illumina.com/documents/documentation/chemistry_documentation/16s/16s-metagenomic-library-prep-guide-15044223-b.pdf), using primers S-D-Bact0341-b-S-17 and S-D-Bact-0785-a-A-21(1) as described previously(2). Final libraries were quantified and analyzed on an Agilent 2100 Bioanalyzer System (Agilent Technologies, Santa Clara, CA), again as described previously(2).

16S amplicon sequencing and analysis

Pooled libraries were paired-sequenced (2x301) on an Illumina MiSeq. Reads were quality-based trimmed and merged using the NGS tool available with CLC Genomic Workbench (Qiagen). A reference-based Operational Taxonomic Unit clustering step using the SILVA v132 database was performed with CLC Microbial Genomics Module v.2.5 (Qiagen), using default parameters for minimum occurrences and chimera crossover cost. Creation of new OTUs was not allowed. Microbial alpha diversity indices including Shannon's, Simpson's, and Observed, were calculated after removal of low depth samples (<5,000 sequences per sample). Inter-sample variability (beta diversity) was calculated using MicrobiomeAnalyst(3, 4). Differences in microbial relative abundances and microbial alpha and beta diversities between groups were calculated using CLC Microbial Genomics Module v.2.5 (Qiagen) and MicrobiomeAnalyst(3, 4). Significance was determined by Wald test and Wilcoxon test. Microbial metabolic pathways

were analyzed using Kyoto Encyclopedia of genes and Genomes (KEGG) collection databases, using PICRUSt2(5).

Supplemental References

1. Klindworth A, Pruesse E, Schweer T, et al. Evaluation of general 16S ribosomal RNA gene PCR primers for classical and next-generation sequencing-based diversity studies. Nucleic Acids Res 2013;41:e1.
2. Leite G, Morales W, Weitsman S, et al. Optimizing Microbiome Sequencing for Small Intestinal Aspirates: Validation of Novel Techniques through the REIMAGINE Study. BMC Microbiol 2019;19:doi:10.1186/s12866-019-1617-1.
3. Dhariwal A, Chong J, Habib S, et al. MicrobiomeAnalyst: a web-based tool for comprehensive statistical, visual and meta-analysis of microbiome data. Nucleic Acids Res 2017;45:W180-W188.
4. Chong J, Liu P, Zhou G, et al. Using MicrobiomeAnalyst for comprehensive statistical, functional, and meta-analysis of microbiome data. Nat Protoc 2020;15:799-821.
5. Douglas GM, Maffei VJ, Zaneveld JR, et al. PICRUSt2 for prediction of metagenome functions. Nat Biotechnol 2020;38:685-688.

Supplemental Figures

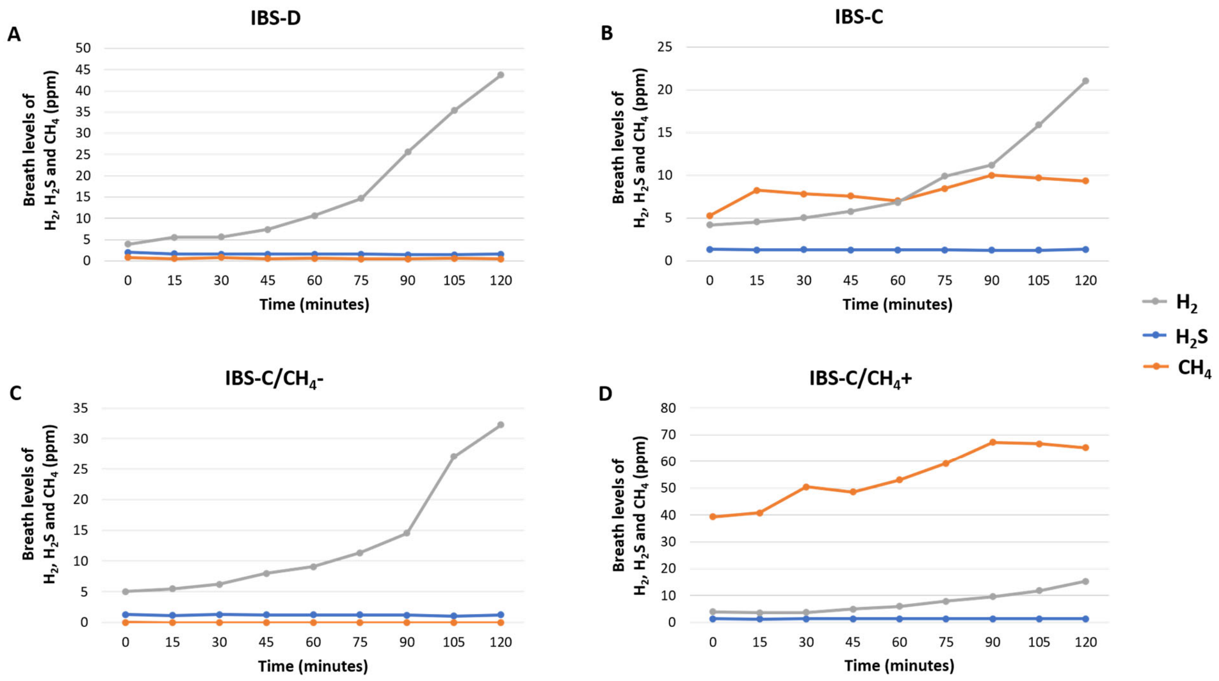

Figure S1. Breath H_2, H_2S and CH_4 levels during the breath test in all subjects. **(A)** IBS-D, **(B)** IBS-C, **(C)** IBS-C/CH_4-, and **(D)** IBS-C/CH_4+ subjects throughout the breath test. All data represent median values.

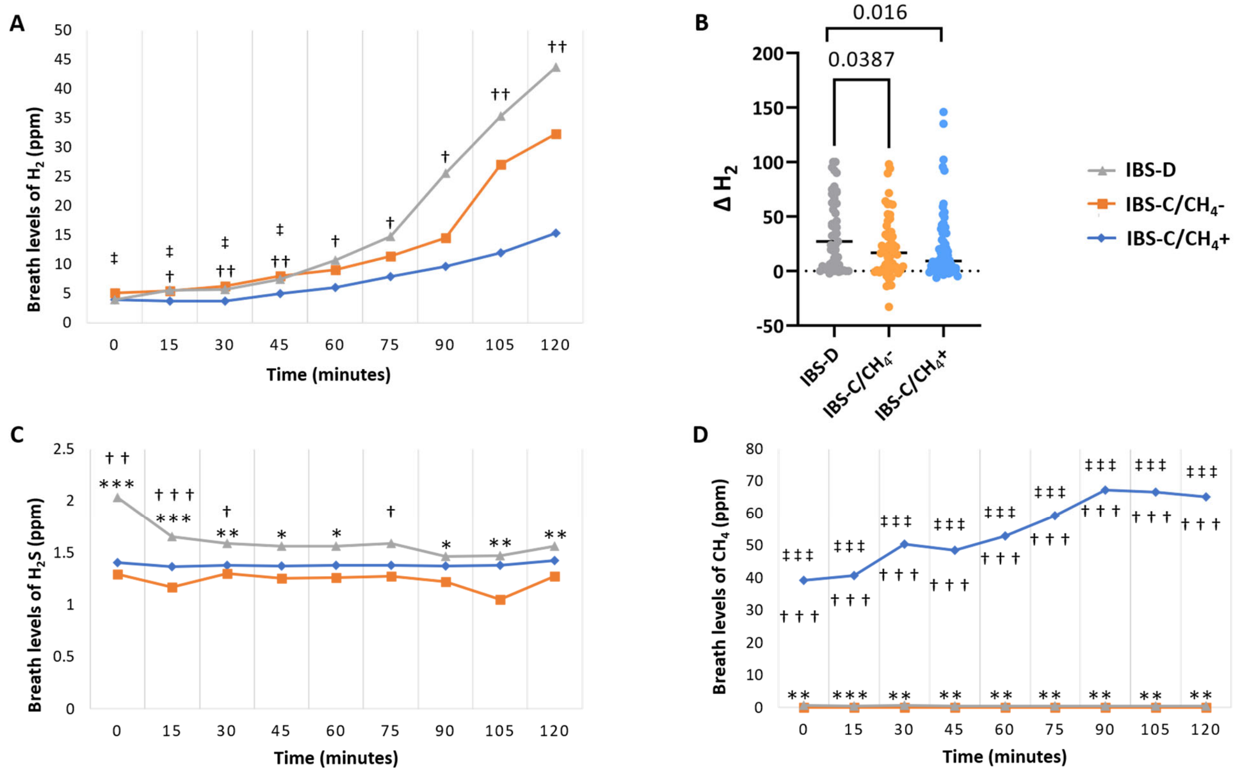

Figure S2. Breath H_2, H_2S and CH_4 levels in IBS-D, IBS-C/CH_4- and IBS-C/CH_4+ subjects throughout the breath test. **(A)** H_2 levels in all groups. **(B)** Scatter plot of delta H_2 levels (comparing levels at 0 and 120 minutes). **(C)** H_2S levels in all groups. **(D)** CH_4 levels in all groups. *P < 0.05; **P < 0.01; ***P < 0.001 IBS-D vs IBS-C/CH_4-; †P < 0.05; ††P < 0.01; †††P < 0.001 IBS-D vs IBS-C/CH_4+; ‡P < 0.05; ‡‡P < 0.01; ‡‡‡P < 0.001 IBS-C/CH_4- vs IBS-C/CH_4+.

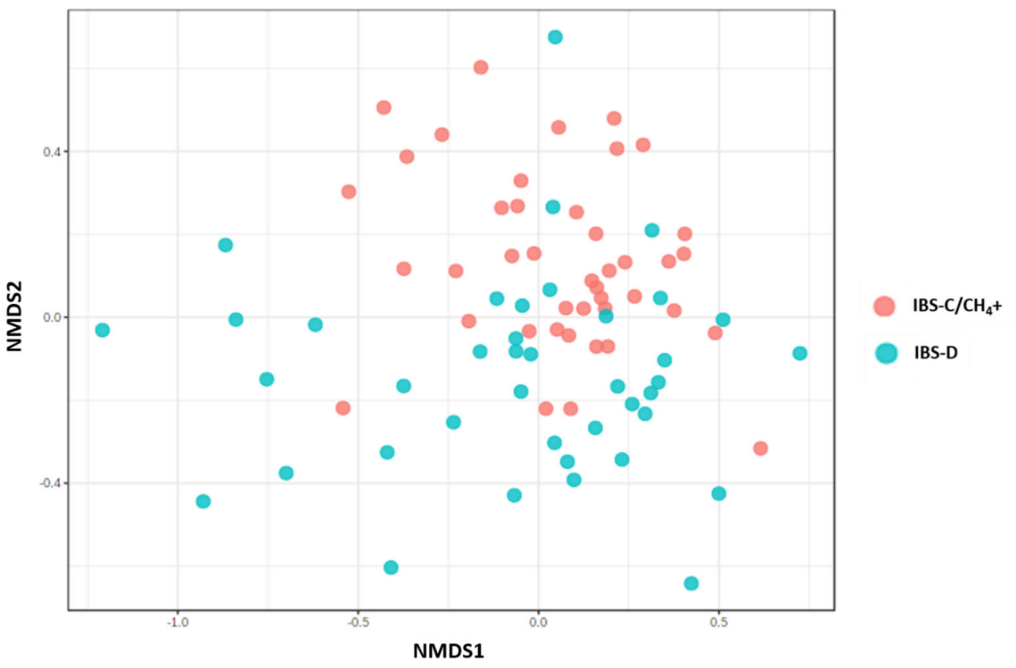

Figure S3. Nonmetric multidimensional scaling (NMDS) ordination of fecal microbiota of IBS-D and IBS-C/CH_4+ subjects. The distance between OTUs was calculated with the Bray-Curtis index. Each circle represents a sample: red - IBS-C/CH_4+, blue - IBS-D. PERMANOVA P-value <0.001.

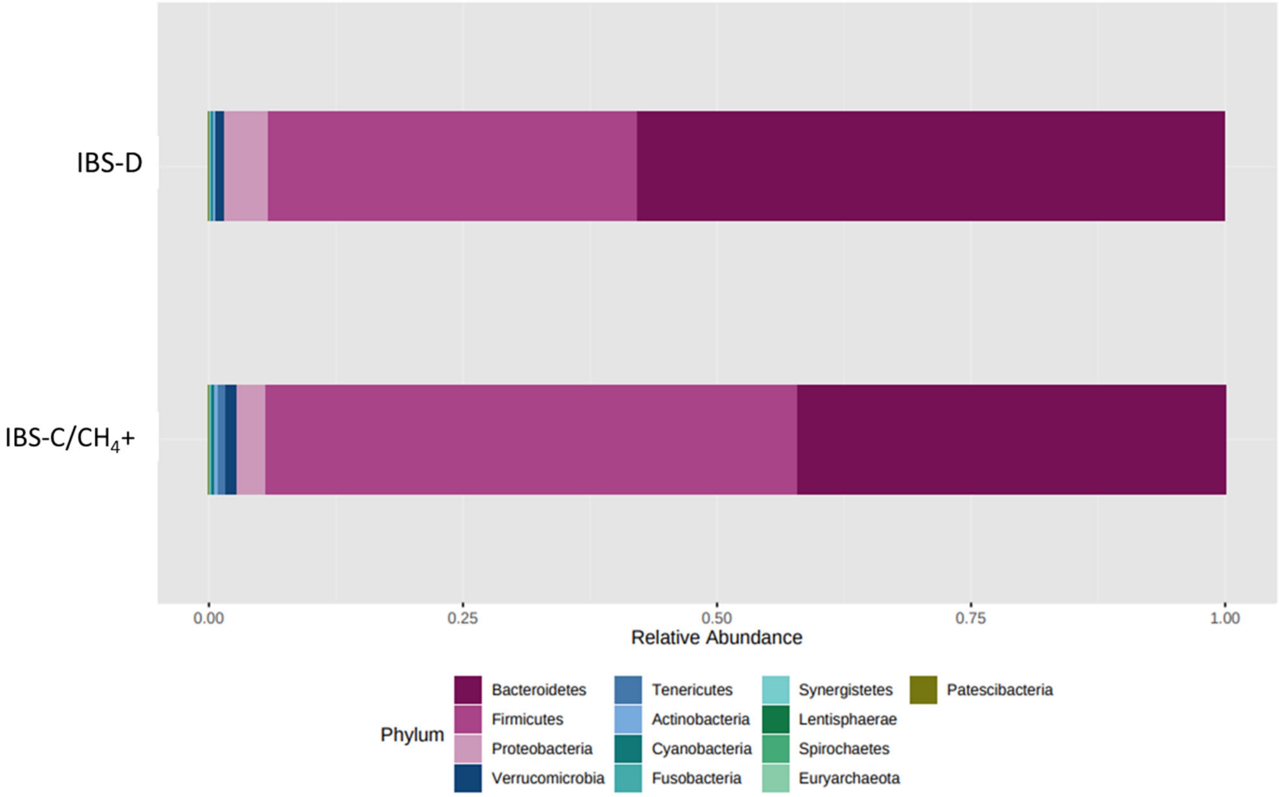

Figure S4. Relative abundances of bacterial phyla in stool samples from IBS-D and IBS/CH_4+ subjects.

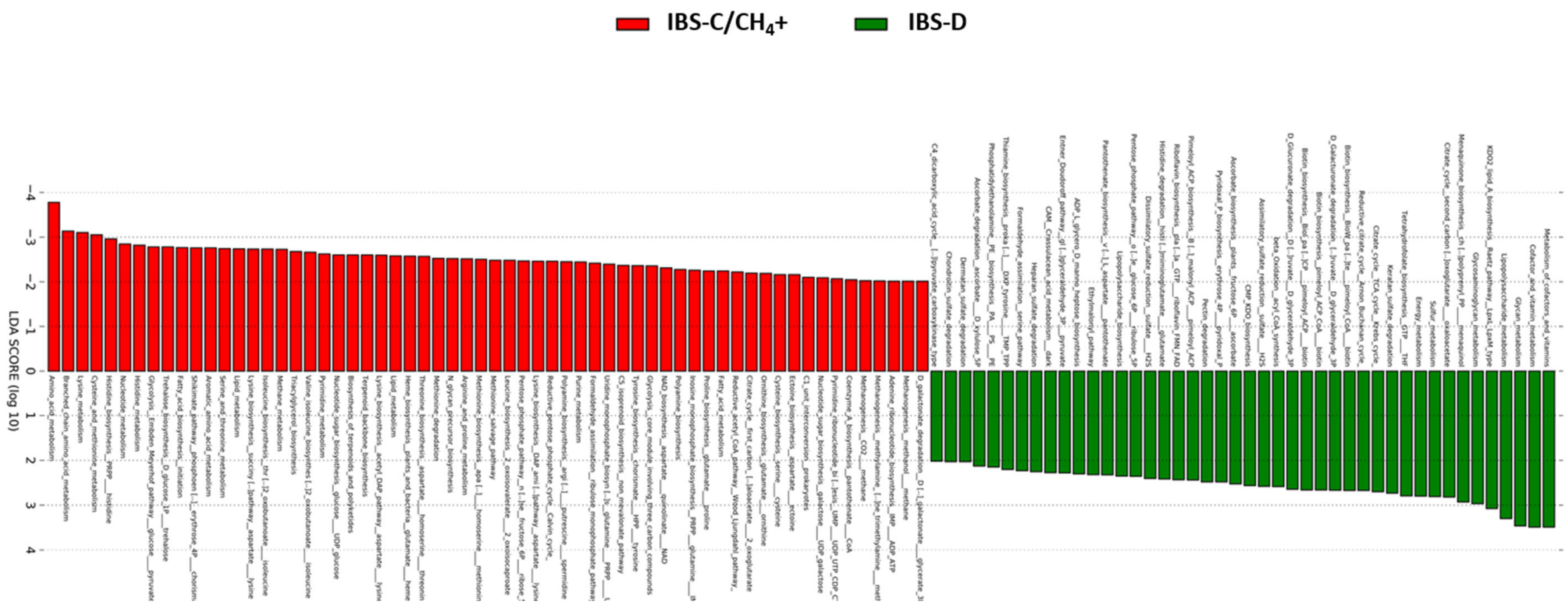

Figure S5. KEGG modules enriched in the stool of subjects with IBS-C/CH_4+ (red) and IBS-D subjects (green).

Table S1: Baseline demographics in IBS-D and IBS-C subjects.

	IBS-D	IBS-C		P-value†
		IBS-C/CH_4-	IBS-C/CH_4+	
**N**	47	58	66	
**Sex (%male)**	36.2	29.3	24.2	0.39
**Age (years ± SD)**	45.2 ± 15.0	43.7 ± 14.0	49.6 ± 10.3*‡	0.04
**BMI (kg/m^2 ± SD)**	23.8 ± 4.9	23.4 ± 4.5	24.4 ± 5.8	0.79

Quantitative data are presented as mean ± standard deviation
†P-value based on three group ANOVA
*IBS-C/CH_4+ vs IBS-D P-value<0.05; ‡IBS-C/CH_4+ vs IBS-C/CH_4- P-value<0.05

Table S2: Spearman's correlation between the absolute abundance of *Methanobrevibacter smithii* in fecal samples and methane and hydrogen levels at each timepoint during the lactulose breath test.

**Breath test time point (minutes)**	**Absolute abundance of *M. smithii* (qPCR)**			
	**Methane levels**		**Hydrogen levels**	
	R	P-value	R	P-value
**0**	0.410	0.003	-0.047	0.750
**15**	0.478	0.001	0.073	0.621
**30**	0.480	<0.0001	0.045	0.759
**45**	0.533	<0.0001	0.100	0.494
**60**	0.540	<0.0001	-0.019	0.897
**75**	0.564	<0.0001	-0.179	0.217
**90**	0.545	<0.0001	-0.265	0.065
**105**	0.560	<0.0001	-0.375	0.008
**120**	0.492	<0.0001	-0.332	0.020

R=Spearman's rank correlation coefficient

Table S3: Differences in relative abundances of microbial taxa in IBS-D vs IBS-C/CH_4+ subjects at the phylum, family and genus levels.

Phylum	IBS-D vs IBS-C/CH_4+			
	Log₂ fold change	Fold change	P-value	FDR p-value
Fusobacteria	5	32.04	3.14E-10	4.43E-09
Spirochaetes	5.91	60.15	5.54E-10	4.43E-09
Euryarchaeota	-3.03	-8.16	4.48E-09	2.39E-08
Tenericutes	-3.47	-11.11	2.45E-07	9.81E-07
Elusimicrobia	-2.9	-7.47	4.91E-04	1.57E-03
Epsilonbacteraeota	2.5	5.65	1.43E-03	3.82E-03
Lentisphaerae	-1.74	-3.35	6.42E-03	0.01
Bacteroidetes	0.47	1.39	8.48E-03	0.02
Synergistetes	-2.09	-4.25	9.21E-03	0.02
Proteobacteria	0.63	1.55	0.01	0.02
Firmicutes	-0.34	-1.27	0.03	0.04
Patescibacteria	-0.81	-1.75	0.11	0.15
Cyanobacteria	-0.71	-1.64	0.29	0.33
Fibrobacteres	-0.82	-1.76	0.29	0.33
Verrucomicrobia	-0.46	-1.37	0.51	0.54
Actinobacteria	0.1	1.07	0.8	0.8

Family	IBS-D vs IBS-C/CH_4+			
	Log_2 fold change	Fold change	P-value	FDR p-value
Pseudomonadaceae	7.26	153.65	0	0
Anaeroplasmataceae	-7.35	-162.8	7.88E-14	3.11E-12
Bacteroidales	6.59	96.43	2.31E-11	6.08E-10
Lactobacillaceae	5	32.03	3.35E-11	6.62E-10
Fusobacteriaceae	5.23	37.44	2.37E-10	3.75E-09
Spirochaetaceae	5.94	61.25	4.49E-10	5.91E-09
Dysgonomonadaceae	5.93	61.06	1.10E-09	1.24E-08
Methanobacteriaceae	-2.79	-6.91	1.63E-07	1.61E-06
Izimaplasmatales	-3.51	-11.35	1.37E-06	1.20E-05
Flavobacteriaceae	-3.84	-14.36	1.87E-06	1.48E-05
Clostridiales vadinBB60 group	-2.29	-4.9	5.78E-06	4.15E-05
Tectona grandis	3.06	8.32	1.43E-05	9.44E-05
Christensenellaceae	-1.91	-3.76	3.23E-05	1.96E-04
Helicobacteraceae	3.25	9.54	2.45E-04	1.38E-03
Clostridium sp. K4410.MGS-306	-2.86	-7.26	3.65E-04	1.92E-03
Enterococcaceae	-2.62	-6.17	4.37E-04	2.16E-03
Coriobacteriales	-2.16	-4.46	4.80E-04	2.23E-03
Elusimicrobiaceae	-2.75	-6.74	8.62E-04	3.78E-03
Bacteroidaceae	0.85	1.81	1.01E-03	4.18E-03
Mollicutes RF39	-2.3	-4.92	1.16E-03	4.60E-03
DTU014	-1.48	-2.8	1.38E-03	5.19E-03
Methanomassiliicoccaceae	-2.08	-4.22	2.55E-03	9.16E-03
Victivallaceae	-1.81	-3.52	3.16E-03	0.01
Caldicoprobacteraceae	-1.92	-3.79	3.81E-03	0.01
Eggerthellaceae	-0.92	-1.9	6.53E-03	0.02
Vibrionaceae	2.01	4.03	0.01	0.03
Defluviitaleaceae	-1.22	-2.34	0.01	0.04
Family XIII	-0.93	-1.91	0.02	0.05
Burkholderiaceae	0.97	1.95	0.03	0.07
Leuconostocaceae	1.67	3.18	0.03	0.08
Peptostreptococcaceae	0.88	1.84	0.04	0.11
Synergistaceae	-1.6	-3.03	0.04	0.11
Bifidobacteriaceae	1.45	2.73	0.06	0.14
Brachyspiraceae	1.54	2.91	0.07	0.16
Prevotellaceae	1.19	2.29	0.1	0.23
Ruminococcaceae	-0.29	-1.23	0.11	0.23
Chloroplast	-0.97	-1.96	0.12	0.26
Peptococcaceae	-0.59	-1.51	0.18	0.37
Family XI-2	-0.84	-1.79	0.19	0.38
Syntrophomonadaceae	-0.99	-1.98	0.21	0.4
Rhodospirillales	0.92	1.89	0.24	0.46
Desulfovibrionaceae	0.54	1.45	0.27	0.51
Atopobiaceae	-0.77	-1.71	0.28	0.52
Coriobacteriales Incertae Sedis	-0.6	-1.52	0.31	0.55
Saccharimonadaceae	-0.54	-1.45	0.31	0.55

Neisseriaceae	-0.76	-1.69	0.32	0.55
Actinomycetaceae	0.65	1.56	0.41	0.62
Marinifilaceae	-0.33	-1.26	0.4	0.62
Muribaculaceae	0.75	1.68	0.41	0.62
Rikenellaceae	-0.25	-1.19	0.38	0.62
Tannerellaceae	-0.3	-1.23	0.45	0.62
Carya cathayensis	0.48	1.4	0.45	0.62
Rubus hybrid cultivar	0.53	1.45	0.38	0.62
Campylobacteraceae	0.55	1.46	0.46	0.62
Fibrobacteraceae	-0.59	-1.51	0.45	0.62
Streptococcaceae	0.33	1.25	0.46	0.62
Eubacteriaceae	-0.47	-1.38	0.4	0.62
Lachnospiraceae	0.1	1.07	0.42	0.62
Micrococcaceae	0.33	1.26	0.51	0.67
Mitochondria	0.39	1.31	0.51	0.67
Acidaminococcaceae	0.41	1.33	0.54	0.7
Leptotrichiaceae	-0.41	-1.32	0.61	0.78
Saccharimonadales	-0.37	-1.29	0.62	0.78
Succinivibrionaceae	-0.5	-1.42	0.64	0.79
Enterobacteriaceae	0.27	1.21	0.67	0.82
Barnesiellaceae	0.24	1.19	0.7	0.83
Pasteurellaceae	0.26	1.19	0.69	0.83
Veillonellaceae	0.19	1.14	0.76	0.88
Coriobacteriaceae	-0.14	-1.1	0.82	0.89
Gaultheria stenophylla	-0.17	-1.13	0.81	0.89
Clostridiaceae 1	-0.14	-1.1	0.79	0.89
vadinBE97	-0.19	-1.14	0.79	0.89
Carnobacteriaceae	0.11	1.08	0.83	0.9
Erysipelotrichaceae	-0.05	-1.03	0.87	0.93
Gastranaerophilales	-0.1	-1.07	0.9	0.94
Akkermansiaceae	0.08	1.06	0.9	0.94
Porphyromonadaceae	0.04	1.03	0.95	0.97
Bacillaceae	0.01	1.01	0.99	0.99
Family XI-1	0.02	1.01	0.98	0.99

Genus	IBS-D vs IBS-C/CH_4+			
	Log$_2$ fold change	Fold change	P-value	FDR p-value
Pseudomonas	7.86	231.96	0.00E+00	0.00E+00
Succinivibrionaceae	7.03	130.41	2.22E-16	3.20E-14
[Eubacterium] oxidoreducens group	-3.43	-10.82	1.65E-14	1.59E-12
Fusobacterium	6.08	67.78	5.21E-14	3.75E-12
[Bacteroides] pectinophilus group	-7.32	-160.27	7.64E-14	4.40E-12
Synergistes	-6.8	-111.26	1.89E-12	9.08E-11
Anaeroplasma	-6.79	-110.58	2.58E-12	1.06E-10
Bacteroidales	6.73	105.9	9.35E-12	3.37E-10
Treponema 2	6.09	68.11	1.10E-10	3.52E-09
Lactobacillus	4.59	24.04	3.77E-10	1.09E-08
Dysgonomonas	5.36	41	1.68E-08	4.40E-07
Sarcina	4.09	17.06	4.55E-07	1.09E-05
Methanobrevibacter	-2.74	-6.68	8.47E-07	1.88E-05
Erysipelotrichaceae UCG-006	4.35	20.46	2.15E-06	4.42E-05
Coprobacillus	3.46	11.04	4.27E-06	8.19E-05
Tectona grandis-1	3.19	9.12	4.68E-06	8.42E-05
Helicobacter	4.04	16.44	7.43E-06	1.26E-04
Izimaplasmatales	-3.21	-9.24	8.67E-06	1.39E-04
Bacteroides	1.15	2.22	9.66E-06	1.41E-04
Veillonella	2.86	7.27	9.77E-06	1.41E-04
GCA-900066225	-2.55	-5.88	1.07E-05	1.46E-04
Flavonifractor	1.99	3.96	1.12E-05	1.47E-04
Cuneatibacter	-3.11	-8.62	1.86E-05	2.33E-04
Ruminococcaceae UCG-011	-2.59	-6	3.11E-05	3.73E-04
CAG-352	-3.41	-10.66	4.19E-05	4.83E-04
Marvinbryantia	-2.04	-4.12	7.80E-05	8.64E-04
Flavobacteriaceae	-3.14	-8.83	1.08E-04	1.12E-03
[Ruminococcus] gnavus group	2.54	5.8	1.07E-04	1.12E-03
Anaerosporobacter	2.83	7.13	1.28E-04	1.23E-03
Oscillospira	-2.31	-4.94	1.26E-04	1.23E-03
Phocea	1.76	3.38	1.89E-04	1.75E-03
Clostridiales vadinBB60 group	-1.9	-3.73	2.12E-04	1.91E-03
Christensenellaceae R-7 group	-1.76	-3.38	3.13E-04	2.65E-03
Merdibacter	-2.26	-4.8	3.12E-04	2.65E-03
Clostridium sp. K4410.MGS-306	-2.67	-6.36	5.69E-04	4.58E-03
Desulfovibrionaceae	3	8.01	5.73E-04	4.58E-03
Enterobacter	-2.91	-7.52	6.01E-04	4.68E-03
Klebsiella	2.94	7.67	6.29E-04	4.77E-03
Megasphaera	2.76	6.76	6.70E-04	4.95E-03
Enterococcus	-2.44	-5.44	9.72E-04	6.82E-03
UC5-1-2E3	-2.06	-4.16	9.54E-04	6.82E-03
Coriobacteriales	-2.06	-4.16	1.01E-03	6.92E-03
Lachnospiraceae	0.92	1.9	1.34E-03	8.49E-03
Romboutsia	1.62	3.07	1.36E-03	8.49E-03

[Clostridium] innocuum group	1.91	3.75	1.32E-03	8.49E-03
Kosakonia	2.79	6.89	1.33E-03	8.49E-03
Rikenella	-2.65	-6.27	1.40E-03	8.58E-03
Mailhella	2.77	6.81	1.43E-03	8.59E-03
Lachnospiraceae FE2018 group	-2.51	-5.71	1.65E-03	9.72E-03
Catenisphaera	-2.55	-5.86	1.92E-03	0.01
Anaerovorax	2.39	5.23	2.03E-03	0.01
Enorma	-2.45	-5.47	2.91E-03	0.02
Elusimicrobium	-2.42	-5.35	3.03E-03	0.02
Sutterella	2.15	4.43	3.03E-03	0.02
Eggerthellaceae	-1.79	-3.47	3.27E-03	0.02
Lachnospiraceae UCG-004	1.42	2.67	3.20E-03	0.02
Moryella	-1.4	-2.64	3.23E-03	0.02
Lactococcus	-1.96	-3.89	3.35E-03	0.02
Victivallaceae	-2.11	-4.32	4.02E-03	0.02
Howardella	1.9	3.73	5.09E-03	0.02
Mitsuokella	2.26	4.8	5.05E-03	0.02
Vibrio	2.18	4.53	5.59E-03	0.03
Ruminococcaceae	-0.82	-1.77	5.90E-03	0.03
Acetitomaculum	-1.89	-3.69	6.52E-03	0.03
Mollicutes RF39	-1.97	-3.91	6.75E-03	0.03
Rikenellaceae RC9 gut group	2.4	5.27	7.01E-03	0.03
Ruminiclostridium 1	-1.62	-3.08	7.11E-03	0.03
Veillonellaceae	-2.06	-4.17	7.51E-03	0.03
Methanomassiliicoccus	-1.91	-3.76	7.69E-03	0.03
Ruminiclostridium	-1.54	-2.9	7.85E-03	0.03
Victivallis	-1.64	-3.12	9.14E-03	0.04
Ruminococcaceae V9D2013 group	-1.87	-3.66	9.81E-03	0.04
Caldicoprobacter	-1.66	-3.17	0.01	0.04
Papillibacter	-1.27	-2.42	0.01	0.04
Herbinix	1.61	3.04	0.01	0.04
DTU014	-1.3	-2.47	0.01	0.04
Prevotella 9	2.08	4.23	0.01	0.05
Ruminococcaceae UCG-007	-1.63	-3.1	0.01	0.05
Defluviitaleaceae UCG-011	-1.24	-2.36	0.01	0.05
Murimonas	-1.8	-3.49	0.01	0.05
Hydrogenoanaerobacterium	-1.42	-2.67	0.01	0.05
Prevotella 2	2.27	4.81	0.02	0.06
[Eubacterium] hallii group	0.84	1.79	0.02	0.06
Tyzzerella 3	1.78	3.44	0.02	0.07
Gordonibacter	-1.33	-2.52	0.02	0.07
Alloprevotella	2.14	4.4	0.02	0.07
[Eubacterium] fissicatena group	1.29	2.45	0.02	0.07
[Eubacterium] nodatum group	-1.1	-2.15	0.03	0.09
Enterobacteriaceae	1.73	3.32	0.03	0.1
Citrobacter	1.46	2.74	0.03	0.1
Proteus	1.82	3.53	0.03	0.1

Catabacter	-1.32	-2.5	0.03	0.11
Bifidobacterium	1.52	2.87	0.04	0.11
Desulfovibrio	1.43	2.7	0.04	0.11
Salmonella	1.77	3.41	0.04	0.11
Lachnospiraceae NK4B4 group	-1.5	-2.83	0.04	0.12
Prevotella 6	-1.57	-2.97	0.04	0.12
Streptococcus	0.93	1.91	0.04	0.12
DTU089	-0.98	-1.98	0.04	0.12
Ruminococcaceae UCG-009	-0.94	-1.92	0.04	0.12
Brachyspira	1.72	3.29	0.04	0.12
Adlercreutzia	-0.98	-1.97	0.05	0.13
Family XIII AD3011 group	-0.99	-1.99	0.05	0.13
Faecalicoccus	-1.59	-3.02	0.04	0.13
Ruminococcaceae UCG-014	-1.22	-2.33	0.05	0.13
CHKCI001	1.36	2.57	0.05	0.13
[Acetivibrio] ethanolgignens group	1.65	3.14	0.05	0.13
Dubosiella	1.65	3.14	0.05	0.13
Senegalimassilia	1.27	2.4	0.05	0.14
Leuconostoc	1.63	3.1	0.05	0.14
Anaerotruncus	-0.84	-1.79	0.06	0.14
Coprococcus 2	1.25	2.37	0.06	0.14
Lachnoclostridium 12	1.54	2.91	0.06	0.15
Lachnoclostridium	0.58	1.5	0.06	0.15
Barnesiellaceae	1.67	3.17	0.06	0.15
CHKCI002	-1.34	-2.52	0.06	0.15
Ruminococcaceae UCG-008	1.37	2.58	0.06	0.16
Asteroleplasma	1.73	3.31	0.07	0.17
Blautia	0.52	1.43	0.07	0.18
Haemophilus	1.13	2.19	0.08	0.19
[Eubacterium] coprostanoligenes group	-0.73	-1.65	0.09	0.2
Anaerofilum	-0.98	-1.97	0.09	0.2
Harryflintia	-0.77	-1.71	0.09	0.2
Prevotella	-1.01	-2.01	0.1	0.22
[Eubacterium] ventriosum group	-0.85	-1.81	0.1	0.22
[Eubacterium] xylanophilum group	-0.78	-1.71	0.1	0.22
CAG-56	1.18	2.26	0.1	0.22
Faecalibacterium	0.62	1.53	0.1	0.23
Weissella	1.24	2.36	0.1	0.23
Robinsoniella	1.24	2.36	0.1	0.23
Agathobacter	0.57	1.49	0.11	0.24
Coprobacter	-1.22	-2.33	0.11	0.25
Sanguibacteroides	1.19	2.28	0.12	0.26
Chloroplast	-0.99	-1.98	0.12	0.26
Atopobiaceae	1.28	2.43	0.13	0.26
Lachnospiraceae UCG-007	1.28	2.43	0.13	0.26
Desulfitibacter	1.29	2.45	0.12	0.26
Comamonas	1.28	2.43	0.13	0.26

Peptococcus	-0.88	-1.84	0.13	0.28
Megamonas	1.17	2.25	0.14	0.28
Escherichia-Shigella	0.94	1.92	0.14	0.29
Christensenellaceae	-0.62	-1.53	0.15	0.3
Tyzzerella 4	1.06	2.09	0.15	0.3
Dorea	0.6	1.51	0.15	0.3
Peptostreptococcaceae	0.76	1.69	0.15	0.3
Peptostreptococcus	-0.91	-1.88	0.15	0.3
Anaerofustis	-0.76	-1.69	0.15	0.3
Lachnospiraceae AC2044 group	-0.86	-1.81	0.15	0.3
Oxalobacter	-0.77	-1.71	0.16	0.32
Ruminococcus 1	-0.58	-1.5	0.17	0.32
Candidatus Soleaferrea	-0.56	-1.47	0.17	0.33
Butyricicoccus	0.5	1.41	0.18	0.34
Pyramidobacter	-1.05	-2.07	0.18	0.34
Anaerovibrio	1.12	2.18	0.18	0.34
Tectona grandis-2	1.12	2.18	0.18	0.34
Prevotellaceae	-1.16	-2.24	0.19	0.35
Ruminiclostridium 5	-0.43	-1.34	0.19	0.35
Subdoligranulum	-0.55	-1.46	0.19	0.35
Erysipelotrichaceae	-0.93	-1.9	0.19	0.35
Holdemania	0.47	1.39	0.19	0.35
Lachnospiraceae FCS020 group	0.65	1.57	0.21	0.37
Carya cathayensis	0.78	1.72	0.21	0.37
Lachnospiraceae UCG-003	1.08	2.11	0.22	0.38
Acidaminococcus	-1.08	-2.11	0.22	0.39
Prevotella 7	1.06	2.08	0.23	0.4
Faecalitalea	-0.75	-1.69	0.23	0.4
Solobacterium	0.88	1.84	0.23	0.4
Phascolarctobacterium	0.83	1.77	0.23	0.4
Rubus hybrid cultivar-1	0.73	1.66	0.24	0.41
Slackia	-0.83	-1.78	0.26	0.44
Pseudocitrobacter	-0.88	-1.84	0.26	0.44
Lachnoclostridium 5	-0.69	-1.61	0.26	0.44
Tyzzerella	0.76	1.7	0.27	0.45
Syntrophomonas	-0.85	-1.8	0.27	0.45
Muribaculaceae	0.95	1.93	0.28	0.46
Cloacibacillus	-0.83	-1.78	0.28	0.46
Anaerostipes	0.47	1.38	0.29	0.47
Actinomyces	0.82	1.77	0.3	0.48
Anaerococcus	0.82	1.77	0.3	0.48
Pseudoflavonifractor	-0.52	-1.43	0.31	0.49
Lachnospiraceae UCG-010	0.43	1.35	0.31	0.49
Rothia	0.54	1.45	0.31	0.49
Ruminococcus 2	-0.58	-1.5	0.31	0.5
Campylobacter	0.72	1.65	0.32	0.5
Lachnospiraceae UCG-006	0.56	1.48	0.32	0.5

Oscillibacter	0.33	1.26	0.32	0.5
Ruminococcaceae UCG-005	-0.46	-1.38	0.32	0.5
Ruminococcaceae UCG-013	0.43	1.35	0.32	0.5
Paraprevotella	0.81	1.75	0.33	0.5
28-4	-0.64	-1.56	0.35	0.53
Fournierella	-0.68	-1.61	0.35	0.53
Candidatus Stoquefichus	0.73	1.66	0.36	0.53
Ruminococcaceae UCG-003	0.53	1.45	0.36	0.54
Lachnospira	0.45	1.37	0.37	0.54
Herbaspirillum	-0.7	-1.63	0.37	0.54
Marinifilaceae	0.63	1.55	0.37	0.55
Erysipelatoclostridium	0.43	1.35	0.37	0.55
Methanosphaera	0.63	1.55	0.38	0.55
Ezakiella	-0.58	-1.49	0.38	0.55
Sellimonas	-0.48	-1.39	0.39	0.56
Coriobacteriales Incertae Sedis	-0.51	-1.42	0.39	0.56
Lactonifactor	0.5	1.42	0.39	0.56
Coprococcus 3	0.45	1.37	0.4	0.56
Prevotellaceae NK3B31 group	-0.77	-1.71	0.4	0.57
Rhodospirillales	0.63	1.54	0.41	0.58
Atopobium	0.65	1.57	0.42	0.59
Ruminiclostridium 9	0.3	1.23	0.43	0.59
Eisenbergiella	-0.4	-1.32	0.44	0.6
Odoribacter	-0.38	-1.3	0.44	0.61
Neisseria	-0.58	-1.49	0.44	0.61
Negativibacillus	0.41	1.32	0.46	0.62
Ruminococcaceae UCG-010	-0.43	-1.35	0.47	0.63
Butyricimonas	0.46	1.37	0.47	0.63
Holdemanella	-0.62	-1.54	0.47	0.63
Clostridium sensu stricto 3	-0.5	-1.42	0.48	0.64
[Ruminococcus] torques group	-0.24	-1.18	0.5	0.67
Erysipelotrichaceae UCG-003	0.39	1.31	0.5	0.67
TM7 phylum sp. oral clone DR034	-0.36	-1.28	0.51	0.67
Peptococcaceae	-0.36	-1.28	0.51	0.68
Dielma	0.32	1.24	0.52	0.68
Shuttleworthia	0.39	1.31	0.52	0.68
[Ruminococcus] gauvreauii group	0.25	1.19	0.53	0.69
Parasutterella	0.37	1.3	0.53	0.69
Ruminiclostridium 6	-0.4	-1.32	0.54	0.7
Granulicatella	0.3	1.23	0.55	0.7
Lachnospiraceae UCG-001	0.3	1.23	0.55	0.7
Catenibacterium	-0.52	-1.43	0.57	0.72
Hungatella	-0.29	-1.23	0.59	0.74
Fibrobacter	-0.41	-1.33	0.59	0.74
Tissierella	-0.41	-1.33	0.59	0.74
Pseudobutyrivibrio	0.4	1.32	0.6	0.75
Allisonella	0.36	1.29	0.6	0.75

Roseburia	-0.17	-1.13	0.61	0.76
Eubacterium	0.32	1.25	0.62	0.76
Fusicatenibacter	-0.22	-1.17	0.62	0.76
vadinBE97	-0.34	-1.27	0.63	0.77
Barnesiella	3.30E-01	1.25	0.64	0.77
Dialister	0.34	1.27	0.64	0.77
Bilophila	2.20E-01	1.16	0.64	0.77
Oribacterium	-3.10E-01	-1.24	0.65	0.78
Eggerthella	-0.22	-1.17	0.66	0.78
Clostridium sensu stricto 1	0.24	1.18	0.66	0.78
Intestinimonas	-0.18	-1.13	0.66	0.78
Rubus hybrid cultivar-2	0.28	1.21	0.66	0.78
Alistipes	-0.14	-1.1	0.68	0.8
UBA1819	0.16	1.12	0.68	0.8
Gemella	0.22	1.16	0.71	0.83
Lachnospiraceae NC2004 group	-0.18	-1.13	0.72	0.83
[Eubacterium] eligens group	-0.21	-1.16	0.72	0.83
Lachnospiraceae UCG-008	-0.13	-1.1	0.73	0.84
[Eubacterium] brachy group	-0.15	-1.11	0.76	0.87
Lachnospiraceae ND3007 group	-0.17	-1.12	0.76	0.87
Lachnospiraceae NK4A136 group	0.13	1.09	0.76	0.87
Erysipelotrichaceae UCG-010	-0.2	-1.15	0.76	0.87
Sneathia	-0.23	-1.17	0.77	0.87
Cronobacter	-0.23	-1.17	0.77	0.87
Acetanaerobacterium	0.15	1.11	0.78	0.88
Bacillus	0.19	1.14	0.8	0.88
Murdochiella	0.19	1.14	0.8	0.88
Lachnospiraceae XPB1014 group	0.21	1.15	0.79	0.88
Saccharimonadales	-0.19	-1.14	0.8	0.88
Parabacteroides	-0.08	-1.06	0.81	0.89
[Eubacterium] ruminantium group	0.18	1.13	0.81	0.89
Ruminococcaceae NK4A214 group	-0.13	-1.1	0.81	0.89
Ruminococcaceae UCG-004	0.1	1.07	0.82	0.89
Porphyromonas	0.13	1.09	0.84	0.9
Butyrivibrio	-0.19	-1.14	0.84	0.9
Incertae Sedis	-0.12	-1.09	0.83	0.9
Libanicoccus	0.13	1.09	0.85	0.91
Asaccharobacter	-0.1	-1.08	0.86	0.91
Angelakisella	-0.12	-1.09	0.85	0.91
Ruminococcaceae UCG-002	-0.08	-1.06	0.86	0.91
Gastranaerophilales	0.14	1.1	0.87	0.91
GCA-900066755	0.11	1.08	0.87	0.91
Olsenella	-0.11	-1.08	0.88	0.92
Terrisporobacter	0.09	1.06	0.88	0.92
Erysipelotrichaceae UCG-004	0.08	1.06	0.9	0.94
Akkermansia	0.08	1.06	0.9	0.94
GCA-900066575	0.04	1.03	0.92	0.95

Succinivibrio	0.1	1.07	0.92	0.95
Turicibacter	-0.05	-1.03	0.93	0.96
Collinsella	0.04	1.03	0.95	0.96
Family XIII UCG-001	-0.03	-1.02	0.95	0.96
Coprococcus 1	-0.02	-1.02	0.97	0.98
Enterorhabdus	-8.03E-03	-1.01	0.99	1
Mogibacterium	-0.01	-1.01	0.99	1
Paraeggerthella	7.43E-03	1.01	0.99	1
Gaultheria stenophylla	2.00E-03	1	1	1
